# Combined posterior and anterior approaches for cervical intradural disc herniation: A case report

**DOI:** 10.37796/2211-8039.1080

**Published:** 2021-03-01

**Authors:** Yu-hsiang Lin, Der-cherng Chen, Chao-hsuan Chen, Hsiang-ming Huang, Der-yang Cho

**Affiliations:** Division of Neurosurgery, China Medical University Hospital, Taichung, Taiwan, ROC

**Keywords:** cervical spine, disc herniation, intradural disc

## Abstract

Intradural disc herniation (IDH) is an extremely rare condition. The authors report the case of a 53-year-old female who had neck and right shoulder pain associated with right-sided hemiparesis and hyperesthesia. Magnetic resonance imaging (MRI) of the cervical spine (C-spine) revealed central mass-like lesions that caused the; compression of the right side of the spinal cord. The posterior surgical approach was used to remove two pieces of IDH. After surgery, the muscle strength in the right upper limb improved from Grade 0/5 to 4+/5 without surgery-related complications. Although there are some reports in literature on the radiologic features of cervical IDH (including the Halo sign, Y-sign, hawk-beak sign, and crumble disc sign), it can be difficult to diagnose radiologically. We present the clinical image of the case along with a review of the literature to remind surgeons to consider IDH as a differential diagnosis when patients are affected by anterior intradural lesions.

## 1. Introduction

Intradural disc herniation (IDH) is an extremely rare condition, comprising less than 0.27% of all disc herniation cases. In the cervical spine, it accounts for only 3% of IDH (as most occur in the lumbar spine[1]) and there are 37 cervical IDH cases reported in literature[2]. Here, we present a case of cervical IDH along with a review of its radiological characteristics.

## 2. Case Presentation

### 2.1. History and Examination

The subject is a 53-year-old female with chronic neck pain that developed with an acute nuchal and right shoulder pain in the span of 5 days. After three days, she developed a right-sided hemiparesis not related to any traumatic event. Neurologic examinations revealed a motor weakness on the right side (Grade 3/5 in elbow extension and flexion, Grade 3/5 in wrist extension and flexion, Grade 0/5 in finger abduction, adduction and grasping, Grade 2/5 in right lower limb). Deep tendon reflexes are 3+ in right triceps reflex and reflexes in right lower limb. Sensory test reveals hypoesthesia below the right C6 dermatome.

**Fig. 1:**
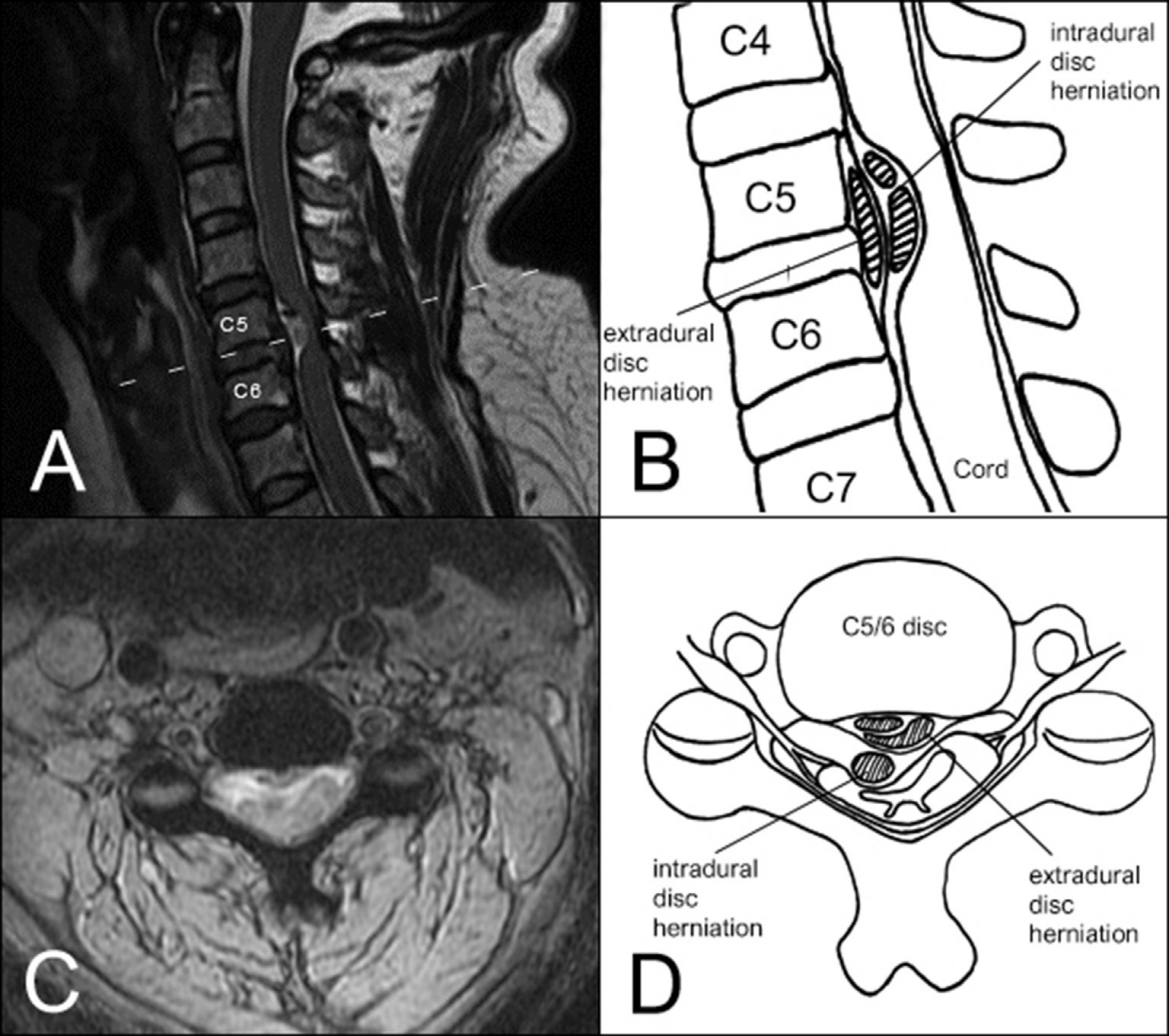
(A) C-spine MR sagittal image shows C5/6 central herniated disc with two intradural extramedullary masses, which are hypointense on both T1WI and T2WI, and surrounded with CSF, assuming a halo feature around the disc fragment. Most neoplasms often tightly attach either to the dura or the spinal cord, and fill the subarachnoid space between them with CSF, making the Halo a strong indicator for. IDH. Two fragments on the sagittal image represent the crumble disc sign. (B) Schematic diagram for 1A (C) Axial cut in Fig. 1A (dash line), revealing that masses compress the cord on the right side (D) Schematic diagram for 1C.

Magnetic resonance imaging (MRI) of the cervical spine disclosed a centrally situated intradural lesion at C5/6, hypointense on both T1WI and T2WI sequences, and surrounded by the cerebrospinal fluid (CSF) (Fig. 1), causing right-sided cord compression. Due to rapid neurological deterioration, we performed a posterior cervical decompression with resection of the intradural mass emergently.

### 2.2. Operation

**Fig. 2:**
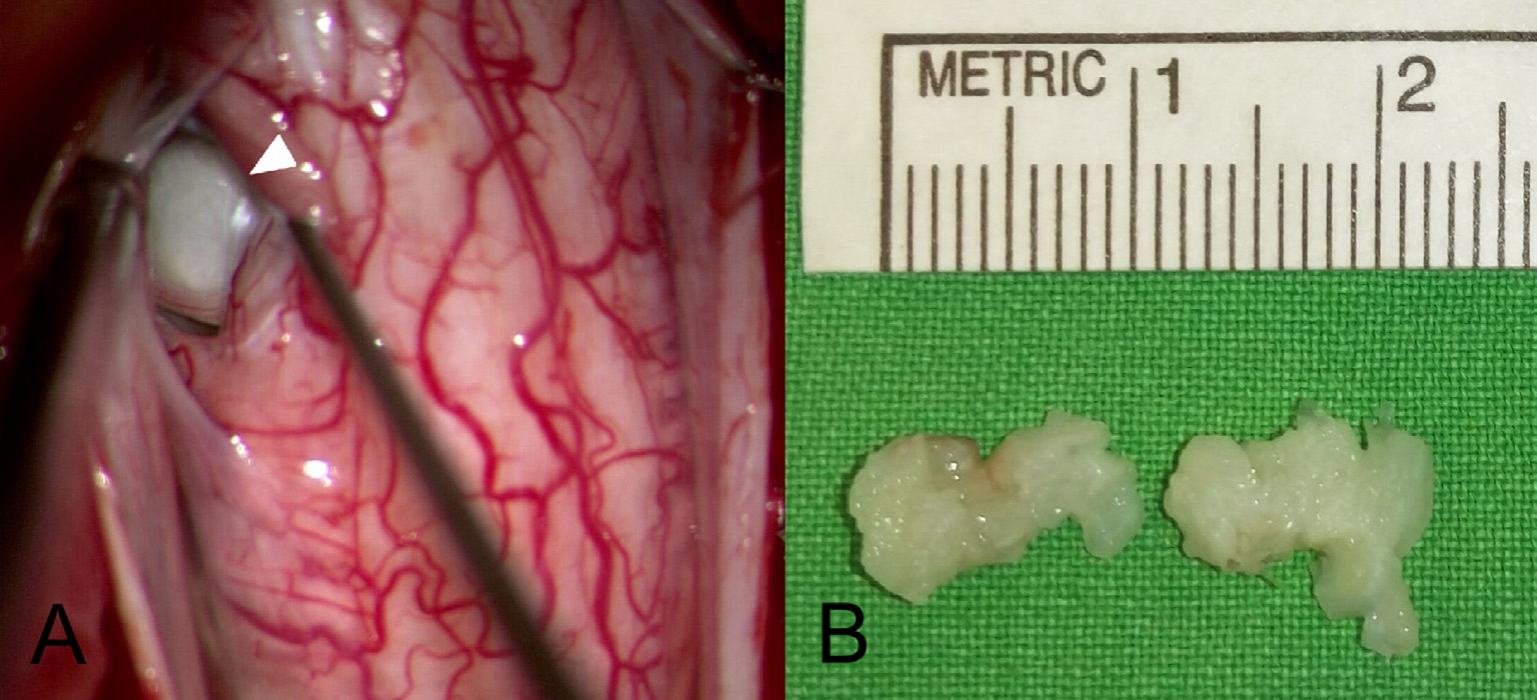
(A) IDH was visualized under a microscopic view via the posterior approach. (B) Two.

**Fig. 3:**
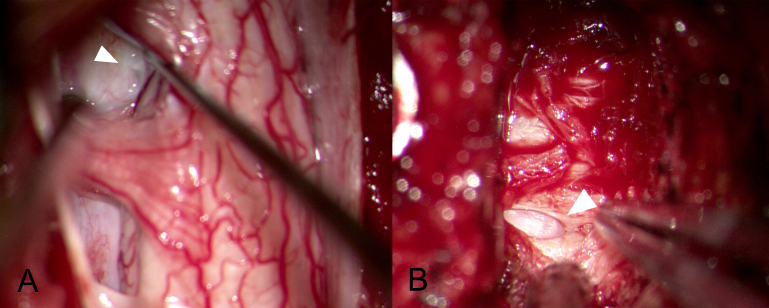
(A) The dural defect (arrow head) was visualized on the right side of C5/6. (B). Longitudinal dural defect with a protruding arachnoid membrane (arrow head) without CSF leakage and severe adhesion between PLL and dura at C5/6 found after anterior discectomy.

A standard prone position for a posterior cervical approach was undertaken. After a laminectomy and durotomy of C4/5/6 was performed, two 10 × 5 × 3 mm ruptured discs (Fig. 2B) were removed from the right posterior lateral aspect through the aid of a surgical microscope. Through the microscope we detected a dural defect on the right side of C5–6 (Fig. 3A). The dura was closed primarily with fibrin glue and a laminoplasty with miniplate was performed.

After a week, a C4/5/6 anterior cervical discectomy was done to treat the extradural disc herniation. Following discectomy, we detected under the microscope a small longitudinal defect at both the posterior longitudinal ligament (PLL) and dura (Fig. 3B), while the arachnoid membrane intact and without CSF leakage.

### 2.3. Postoperative Course

After surgery, the patient’s hypesthesia symptoms improved. After a 24-month follow-up, motor strength was restored to Grade 4+/5 with mild paresthesia. The patient was able to walk with a cane and self-care with the assistance of tools. No surgery-related complications occurred.

## 3. Discussion

**Fig. 4:**
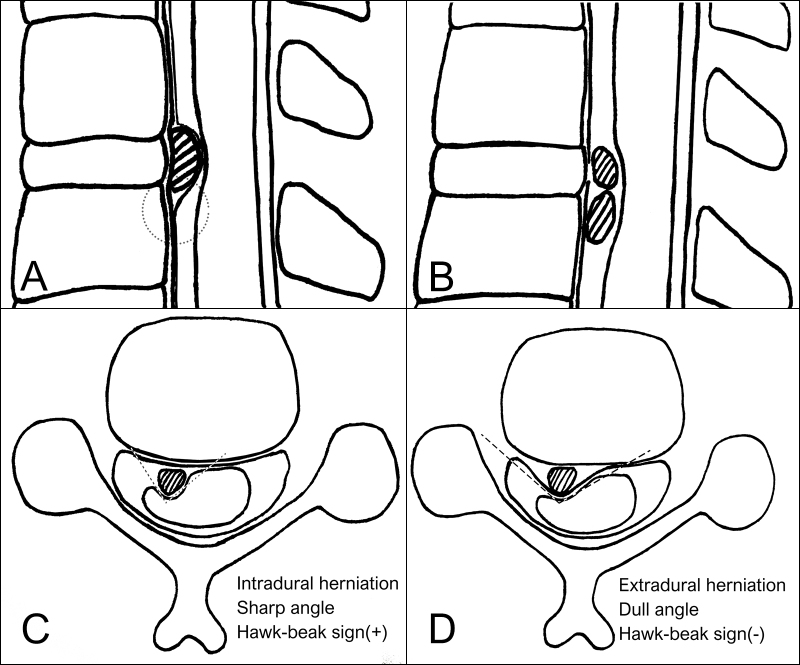
(A) For an intradural extra-arachnoid disc herniation, the dura and arachnoid membrane are separated by the intradural disc fragment and CSF, which resulted in a Y-shape (dash circle). Usually neoplasms occur in the subarachnoid space while the arachnoid membrane tightly attaches to the dura. (B) Multiple fragments of IDH 12 represent the Crumble disc sign, while neoplasms usually occur as a solitary lesion. (C, D) A sharp herniated disc angle, like a hawk-beak, represents IDH. Because the disc fragment is confined to the dura, a dull angle appears in the extradural disc herniation.

Although some reports in current literature have described the radiological features of IDH, such as the Halo sign[3], Y-sign[4], hawk-beak sign[5] and crumble disc sign[6], it remains difficult to diagnose radiologically before the operation. The Halo and crumble signs are evident in this case (Fig. 1). The Halo sign is thought to be a ruptured hypointense disc surrounded by hyperintense CSF on T2WI. The Y-sign (Fig. 4A) might be related to an intradural extra-arachnoid herniation, causing the accumulation of CSF between the separated dura and arachnoid membrane. Both the Halo sign and Y-sign are strong indicators for IDH. The hawk-beak sign (Fig. 4C and D) represents an intradural extra-arachnoid disc herniation without CSF accumulation, with the sharp angle resulting from the disc fragment as it appears on the MRI. The crumble disc sign (Fig. 4B) represents multiple nodular fragments. All these radiological characteristics help in the preoperative diagnosis of IDH.

Pan et al. state that anterior decompression leads to a better outcome since the posterior approach makes it hard to achieve complete decompression [7]. However, Arunprasad Gunasekaran et al.[2] advocate that the posterior approach along with the separation of the denticulate ligament and a gentle mobilization of the cord results in a successful outcome. In this case, we chose the posterior approach, allowing the preservation of C5 vertebral body.

The pathophysiology of the IDH is still unclear and thought to be connected to chronic inflammation of the PLL and the PLL adhered to dura. Once the disc herniated, disc fragment goes through both PLL and dura due to the adhesion [2, 7].

## 4. Conclusion

In conclusion, we reported this case and reviewed the literature to remind surgeons that IDH should be considered as a differential diagnosis when patients present with anterior intradural lesions. We believe that the best choice is the combination of the two approaches as the anterior discectomy may not achieve complete decompression or mass removal while the anterior corpectomy may possess higher risks for anterior instrument failure and plate complication.
